# What is memory? The present state of the engram

**DOI:** 10.1186/s12915-016-0261-6

**Published:** 2016-05-19

**Authors:** Mu-ming Poo, Michele Pignatelli, Tomás J. Ryan, Susumu Tonegawa, Tobias Bonhoeffer, Kelsey C. Martin, Andrii Rudenko, Li-Huei Tsai, Richard W. Tsien, Gord Fishell, Caitlin Mullins, J. Tiago Gonçalves, Matthew Shtrahman, Stephen T. Johnston, Fred H. Gage, Yang Dan, John Long, György Buzsáki, Charles Stevens

**Affiliations:** Institute of Neuroscience, Chinese Academy of Sciences, Shanghai, China; RIKEN-MIT Center for Neural Circuit Genetics at the Picower Institute for Learning and Memory, Department of Biology and Department of Brain and Cognitive Sciences, Massachusetts Institute of Technology, Cambridge, MA 02139 USA; Howard Hughes Medical Institute, Massachusetts Institute of Technology, Cambridge, MA 02139 USA; MPI of Neurobiology, Munich-Martinsried, Germany; Department of Biological Chemistry and Department of Psychiatry and Biobehavioral Studies, David Geffen School of Medicine, BSRB 390B, 615 Charles E. Young Dr. South, University of California, Los Angeles, CA 90095 USA; Picower Institute for Learning and Memory, Department of Brain and Cognitive Sciences, Massachusetts Institute of Technology, Cambridge, MA 02139 USA; The Neuroscience Institute, School of Medicine and Center for Neural Science, New York University, New York, NY 10016 USA; Salk Institute for Biological Studies, Laboratory of Genetics, 10010 N. Torrey Pines Road, La Jolla, CA 92037 USA; HHMI, Department of Molecular and Cell Biology, University of California, Berkeley, USA

## Abstract

The mechanism of memory remains one of the great unsolved problems of biology. Grappling with the question more than a hundred years ago, the German zoologist Richard Semon formulated the concept of the engram, lasting connections in the brain that result from simultaneous “excitations”, whose precise physical nature and consequences were out of reach of the biology of his day. Neuroscientists now have the knowledge and tools to tackle this question, however, and this Forum brings together leading contemporary views on the mechanisms of memory and what the engram means today.

## The cellular basis of memory

### Mu-ming Poo

Neurobiological studies of memory over the past century have progressed along two relatively independent lines of inquiry: the top-down approach examines the animal’s behaviors associated with memory acquisition, consolidation, and retrieval, as well as the brain regions underlying these processes, whereas the bottom-up approach explores the cellular and circuit mechanisms of memory encoding and storage by examining the patterns of neuronal firing and the efficacy of synaptic transmission. In his monumental treatise [1] *The Organization of Behavior* (1949), Donald Hebb made a bold attempt to link these two lines of inquiry by postulating that perceptual memory resides in specific “cell assemblies” formed by the strengthening of interneuronal connections due to correlated activities during memory acquisition. The discovery of activity-induced long-term potentiation (LTP) and long-term depression (LTD) of central synapses in the 1970s and 80s further sparked the interest of a whole generation of neurobiologists in studying synaptic plasticity and its relationship to memory. There is now general consensus that persistent modification of the synaptic strength via LTP and LTD of pre-existing connections represents a primary mechanism for the formation of memory engrams. In addition, LTP and LTD could also lead to the formation of new and elimination of old synapses and thus changes in structural connectivity in the brain. Indeed, early development of neural circuits, whereby neural activity sculpts synaptic connectivity [2], depends on processes similar to that associated with LTP and LTD in the adult brain and could be considered as the imprinting of memory engrams generated by early experience.

In this Forum, a group of experts on the cellular mechanisms of memory were invited to present their views on “what is memory”, including where and how memory engrams are stored, consolidated, and retrieved. Drawing on an elegant set of studies, Michele Pignatelli, Tomás Ryan, and Susumu Tonegawa illustrate how recently developed techniques to tag and manipulate neurons have begun to establish a causal link between neuronal activity, persistent synaptic changes, and an animal’s memory-associated behaviors. The theme of persistent synaptic changes and their causal role in memory is taken up by Tobias Bonhoeffer, who summarizes the evidence that dendritic spines, where excitatory synapses are located, represent the basic cellular unit for memory; long-term memory is stored in a set of spines that are formed or modified during learning and these changes may persist throughout the animal’s life.

Based on the findings of activity-induced transcriptional activation and synapse-specific local translation of proteins, Kelsey Martin expands on the idea that the basic building block of memory is the synapse, where both pre- and postsynaptic elements together with associated glial processes form an integral unit with an individual identity and distinct “neighborhood”. Andrii Rudenko and Li-Huei Tsai redirect attention to the nuclei of engram cells, discussing the evidence that epigenetic alterations of the neurons activated during memory acquisition may be involved in the long-term retention of memory. They propose that such epigenetic modification represents a priming event during the initial phase of memory formation; memory retrieval would then trigger the expression of the primed genes, leading to protein synthesis and synaptic modification at individual synaptic units.

Depending on the availability of cellular resources, immediate modifications (LTP and LTD) and long-term turnover (formation and elimination) of individual synaptic units are bound to influence other units on the same postsynaptic cell. Richard Tsien, Gord Fishell, and Caitlin Mullins focus on the important issue of lateral synaptic interaction and redistribution of synaptic strength associated with LTP and LTD, from the point of view of cellular homeostasis as well as the normalization and signal-to-noise ratio of neuronal activities, and propose a conceptual scheme to address the underlying mechanisms.

The hippocampus is unique in being a key brain region for memory formation and a region in which adult neurogenesis occurs. Associated with hippocampus-dependent spatial memory, Tiago Gonçalves, Matthew Shtrahman, Stephen Johnston, and Fred Gage discuss an intriguing new dimension in the cellular mechanisms of memory formation, whereby continuous addition of newborn dentate gyrus neurons in the adult hippocampus, with their enhanced synaptic plasticity, may contribute significantly to establishing the engram for spatial memory.

As proposed by David Marr in his model of hippocampus-dependent memory [3] and supported by many experimental and clinical studies, episodic memories are transferred after acquisition from the hippocampus to the neocortex for long-term storage. The mechanisms underlying the transfer and consolidation of spatial memory are discussed by John Long and György Buzsaki in the context of hippocampal and entorhinal sharp wave-ripples. These activity patterns occur during sleep or non-attentive brain states and are replays of neuronal firing sequences triggered by recent experience, for example they can be temporally compressed replayed versions of the sequential neuronal firing seen as the animal traverses through a particular environment. As discussed by myself and Yang Dan, although spike timing-dependent plasticity could offer a synaptic mechanism for storing sequence information with intervals up to a few hundreds of milliseconds, it remains largely unknown how neural circuits store and recall the temporal sequence of information up to seconds and longer, periods often associated with episodic memory. A compression of the temporal sequence of events such as occurs during sharp wave-ripples in the hippocampus and neocortex offers a potential solution.

The contributing articles of this Forum reflect the tremendous progress made in our understanding of the cellular building blocks of memory. There is a clear consensus on where the memory engram is stored—specific assemblies of synapses activated or formed during memory acquisition—and a substantial body of knowledge on how the engram is generated and maintained in the brain. However, knowing the building blocks and their properties is far from understanding the architecture of the “memory palace”. As Charles Stevens indicates in his epilogue, and the readers will soon discover, many new territories are now open for exploration.

## Engram cell connectivity as a substrate for memory storage

### Michele Pignatelli, Tomás J. Ryan, and Susumu Tonegawa

The storage of information refers to the systematic process of collecting and cataloging data so that they can be retrieved on request.

One of the most enlightening conceptualizations of the neural representation of stored memory information was developed by Richard Semon, who conceived the Engram Theory, a theory of memory traces [4]. According to this theory, as fortified by contemporary knowledge, learning activates a small ensemble of brain cells, inducing in these cells persistent physical/chemical changes. In addition, reactivation of these cells by relevant recall cues results in retrieval of the specific memory. The theory poses an important question: what is the nature of the persistent changes?

In his seminal book published in 1949, Donald Hebb proposed a mechanism based on synaptic plasticity as a substrate of memory [1]. With an example of two cells connected by an excitatory synapse, if the activation of one cell leads to the activation of the second one, the connection between the two cells is reinforced, a postulate that has been confirmed experimentally [5–8]. The increase in connectivity strength within a diffuse group of cells in a more complex feedforward circuit results in the emergence of an engram cell ensemble.

The systematic dissection of the molecular mechanisms involved in synaptic plasticity has revealed that the cascade of events underlying the plastic changes requires two distinct phases [9, 10]. In the encoding phase, also known as early long-term potentiation (E-LTP), an increase in intracellular Ca^2+^ concentration mediated by post-synaptic NMDA receptors elicits a change in synaptic weight by increasing the insertion and the conductance of AMPA receptors [11]. The dendritic spines that support the post-synaptic machinery rapidly increase in number [12]. In a second phase lasting a few hours after the initial encoding period, the increased synaptic weight is maintained by a protein synthesis-dependent process known as cellular consolidation, during which the steady state synthesis of AMPA receptors is shifted to a higher level. This second phase is known as late LTP (L-LTP) [9, 10] and is sensitive to protein synthesis inhibitors (PSI).

To date, memory storage has mainly been investigated by pharmacological or molecular manipulation and by correlating synaptic changes with the strength of memory recall. Only recently has it become possible to specifically tag cells activated by learning. The demonstration that these cells are part of a memory engram ensemble was provided by a series of “optogenetic” experiments where a learning-induced tagging strategy was employed to express channelrhodopsin [13] in a small population of learning-activated cells [14, 15]. The opsin allowed for the artificial light-induced reactivation of the cellular population labeled during learning and resulted in memory retrieval. The same tagging strategy also verified the reactivation of tagged cells upon presentation of retrieval cues.

Tagging “engram” cells offers a straightforward opportunity to investigate the nature of the persistent changes that occurred in these cells in response to learning. In a recent study [16], “engram cells” were compared to “non engram cells” (non-tagged cells) by ex vivo patch clamp recordings after contextual fear conditioning (CFC). Engram cells displayed changes in synaptic weight typical of LTP such as high current amplitude, insertion of AMPA receptors, high spontaneous excitatory post-synaptic current frequency and amplitude, and increased dendritic spine density. These changes were blocked by the systemic injection of PSI specifically within the consolidation window. Therefore, it is now clear that cells recruited by learning display synaptic changes typical of LTP and are reactivated by retrieval cues, and their reactivation can elicit memory recall. Remarkably, however, protein synthesis-dependent L-LTP seems to be dispensable for memory storage because direct optogenetic activation of the engram cells in PSI-injected mice elicited full memory recall under a variety of conditions.

#### If L-LTP is dispensable for memory storage, what mechanism is responsible?

An integral memory engram may consist of preferential connectivity between engram cell ensembles distributed across multiple brain regions. In the same report [16] it was shown ex vivo that engram cells from the dentate gyrus established preferential connections with engram cells in the downstream hippocampal CA3 region in a feedforward excitatory engram cell circuit. Remarkably, this preferential connectivity was maintained in mice rendered amnesic by treatment with PSI within the consolidation window, suggesting that memory storage may survive retrograde amnesia in the form of a neural connectivity pattern. Indeed, optogenetic stimulation of DG engram cells in vivo elicited similar cellular reactivation patterns not only in the CA3 region but also in the amygdala for both control and amnesic groups, thus confirming the persistence of engram cell connectivity. These observations support the concept of preferential connectivity of engram cell ensembles distributed across multiple brain regions, which is established during learning and persists despite disruption of consolidation and thereby provides a lasting substrate for memory storage. These data also suggest that the synaptic potentiation observed in consolidated engram cells is necessary for memory retrievability and not for storage [17]. While regulation of synaptic weight provides a scalar quantity to control information retrieval, synaptic connectivity holds the information specificity. This is because synapses that are activated during the encoding stage will dictate the eventual pattern of cellular connectivity of the upstream and downstream engram cell ensembles. This notion is compatible with the broad view that synapses are the basic units of information storage (see Bonhoeffer, this Forum).

#### How would the specific connectivity pattern between engram cell ensembles be formed by specific learning?

Neural connections are formed during development and certain circuits hold the innate capability to elicit complex behavioral reactions in response to specific perceptual cues [18]. However, this does not seem to be the case in the hippocampal formation because inactivation of the downstream CA1 region before CFC results in anterograde amnesia which cannot be bypassed by direct optogenetic stimulation of DG engram cells [16]. Thus, the memory circuit is not configured under anterograde amnesic conditions and is not, therefore, genetically determined but requires hippocampal activity during memory encoding. As reported by Ryan et al. [16], learning-induced changes in connectivity patterns are insensitive to PSI. So is E-LTP [9, 10] and this early phase of plasticity may provide a framework to investigate the formation of new connections. For instance, blocking NMDA receptor function should impair the emergence of learning-induced connectivity patterns.

During the induction of LTP, existing connections can be potentiated [19] but new connections can also emerge [20]. A hypothetical way this might happen is through the activation of silent synaptic connections [21]. These synapses expressing only NMDA receptors and not AMPA receptors can become unsilenced through AMPA receptor insertion, a mechanism that could, in principle, support the formation of learning-induced changes in engram cell connectivity. Another possibility is that local dendritic protein synthesis contributes to the rapid synapse formation on engram cells, independently of its role in synaptic potentiation (see Martin, this Forum).

#### What then maintains the learning-induced engram cell connectivity that is initiated during encoding?

**Fig. 1:**
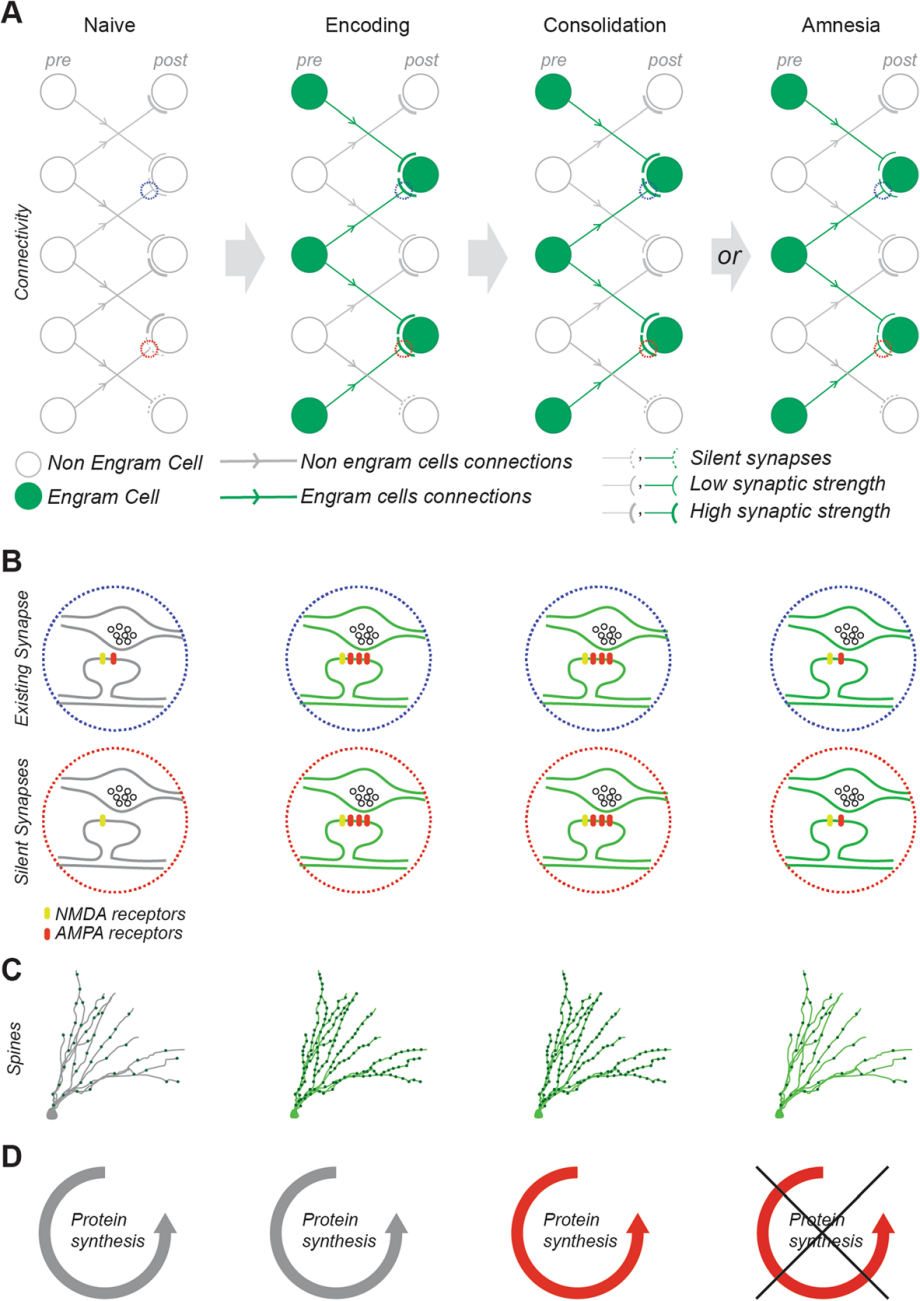
Synaptic connectivity between engram cells as a mechanism for memory storage. **a** Cellular connectivity in a feedforward excitatory circuit, **b** synaptic configuration, **c** dendritic spine density, and **d** protein synthesis state, shown in a naïve circuit, a circuit during encoding, a circuit after consolidation, or a circuit in an amnesic condition. Engram circuit, cells, and synapses are displayed in *green*, non-engram in *gray*. In the naïve state, the circuit displays a variety of synaptic patterns, including strong (*thick gray lines*) and weak synapses (*thin gray lines*) as well as silent synapses (*dotted lines*) exclusively expressing NMDA receptors. During encoding, a network of engram cells is recruited. The preferential connection between engram cells occurs either by potentiation of existing connections (*blue dotted circles*) or by unsilencing synapses (*red dotted circles*). A spine density increase supports the synaptic changes. During consolidation, the steady state synthesis of AMPA receptors is shifted to a higher level and the disruption of consolidation with protein synthesis inhibitors (PSI) results in retrograde amnesia. However, during PSI-induced amnesia, memory storage persists within an engram-specific set of weak synaptic connections

Although learning-induced synaptic potentiation would be suppressed by PSI, the new connectivity pattern could persist through unsilenced synaptic connections of basal unpotentiated strength (Fig. 1). Consistent with this perspective, it has been recently shown that optogenetically induced long-term depression (LTD) of amygdala cells impaired existing conditioned fear responses but subsequent optogenetically induced LTP of the same cells could restore optogenetic cue-evoked recall of the fear memory [22].

The ability to tag cells activated by learning has opened up new horizons in the investigation of memory by revealing the role of the engram-specific connectivity in the storage of information.

## Spines and synapses as basic elements of memory storage

### Tobias Bonhoeffer

In his seminal work, Donald Hebb [1] proposed that the basic mechanism by which memories are stored in the brain is the enhancement of synaptic strength and, in connection with that, morphological changes of the respective synaptic contacts. In other words he proposed that synapses and not cells are the basic building blocks of memory, from a theoretical perspective a reasonable suggestion as there are approximately 10,000–100,000 times more synapses in the brain than neurons.

By now it is well established that morphological changes at the synaptic level occur in conjunction with stimuli that are thought to mimic learning events. In vitro experiments [12, 23] have shown that long-term potentiation results in the addition of dendritic spines, tiny protrusions which harbor synaptic contacts. Tens of thousands of these spines decorate the dendrites of most excitatory cells in the hippocampus and the neocortex. And indeed, further studies showed that spines not only come and go but also change their shape during putative learning events [19], a suggestion that had been put forward in a purely theoretical paper by Francis Crick [24]. So, it is well established that spines emerge, disappear, and change with cellular events thought to underlie learning processes. But is this merely a correlation or are there ways towards showing that these events really lie at the basis of learning and memory storage in the brain? Recent experiments have made substantial progress in this respect.

The first study that made a clear case that spines are important for the long-term storage of information was done in the visual cortex of mice [25]. In the visual cortex it is well known that synaptic connections are established or modified with changes in visual experience, like the temporary closure of one eye. These plastic changes are often used as a proxy for what happens during memory formation since they share key features: it is, for instance, a universally accepted fact in memory research that information that has been acquired early in life can be learned much more easily a second time, even if it had been “completely forgotten” in the meantime. This effect has been called “savings” [26] and is a hallmark of most memory processes. It has been shown that the same effect occurs in the visual system [27]. Mice were monocularly deprived for a couple of days early in life so that the visual system adapted to this change of the visual environment. Subsequently, animals were subjected to normal vision again so that their visual cortex reverted to normal function. If monocular deprivation was then performed a second time, much later in life, when normally this procedure has only a very limited effect (if any), substantial adaptation still takes place because of the early experience that the animal has had. Importantly, this savings effect could be related to new spines that emerged during the first plasticity episode and persisted [25]. The fact that there was no growth of additional spines during the second plasticity period, while the functional adaptation occurred much faster and more reliably, suggests that the persistent spines facilitate the second adaptation [25]. Therefore, these spines serve to “remember” the previous sensory experiences the animals had. Two subsequent studies [28, 29] further bolstered the case by showing that also in the motor cortex the generation of new spines forms the basis of learning motor tasks of different sorts. Interestingly, in one of these studies [28] it was also found that relearning a task occurred faster and did not involve the generation of new spines, again arguing for persistent spines “memorizing” specific motor tasks. Furthermore, this study demonstrated that learning different tasks involves different sets of spines, providing a strong argument for spines and not cells being the relevant entity for information storage in the brain.

These three papers were among the first to make a strong case for a causal relationship between new (or changing) spines and learning or information storage in the brain. Subsequently, a number of studies further strengthened this hypothesis. Some of them used fear conditioning to show that also in this paradigm learning is paralleled by structural changes: fear extinction and fear conditioning are marked by the generation or removal of spines in the frontal association cortex [30] and the auditory cortex [31]. One particularly interesting finding in this context is that extinction induces appearance of spines that were eliminated upon the original fear conditioning to the same stimulus but not to a distinct conditioned stimulus, suggesting that the spines are again specifically associated with extinction of one specific association [30]. Interestingly, also in a completely different animal model—song learning in zebra finches—it was shown that new spines are generated in the forebrain nucleus HVC when an animal learns a new song from a tutor [32].

Finally, what about the experiment that has long been on the agenda [33], namely to specifically ablate spines that have been generated during learning? If the above interpretations are true, spine ablation should lead to forgetting of the information that was learned when the new spines were generated. First important strides in that direction have been made in a recent experiment by the group of Haruo Kasai [34], who specifically labeled spines that were generated at a particular time window immediately after learning. When these spines were later ablated or at least reduced in size, the animal indeed forgot the previously learned information. The learning paradigm is so far relatively simple (rotarod learning) but it provides a very nice indication that the generation of new spines or their enlargement is truly causal at least in some forms of learning.

Taken together, there is now considerable evidence from different species as well as from different learning paradigms that spines, and thus synapses, change when an animal learns. Furthermore, there are convincing indications that the maintenance of previously established structural connections on the level of dendritic spines explains the memory phenomenon of savings. Finally, if spines are ablated, an animal forgets what it has learned through the addition of new or stronger spine synapses. All of these experiments seem to point strongly towards the notion that spines or synapses (and not entire cells) may be the smallest unit of memory storage in the brain and it may, therefore, be most appropriate to say that the “engram” of a memory is laid down in the set of spines or synapses that are changed when specific information is stored. This is of course not to say that engrams are not visible on the level of single cells (see preceding contribution to this Forum by Pignatelli, Ryan, and Tonegawa); after all, the activity of cells is determined by the complement of their synapses. Yet, the finest resolution of the engram may only become apparent if one truly considers everything on the basis of the pattern of synapses or spines which are changed during a particular memory event.

## A cell biologist’s view of memory: revisiting the neuron doctrine

### Kelsey C. Martin

Over a century ago, the anatomist Ramon y Cajal used the Golgi staining method to visualize individual cells in the brain. He observed that the brain was composed of discrete cells rather than of a “reticular network” of interconnected cells (as was commonly believed at the time). Cajal’s observations provided critical support for the neuron doctrine, which postulates that the neuron is the central unit of the brain. At the same time, his drawings revealed the beautiful complexity, polarity, and compartmentalization of neurons: cell bodies elaborating axonal and dendritic processes, forming up to thousands of synapses with one another. With a remarkable amount of prescience, Ramon y Cajal also speculated that memories were stored as increases in the numbers of connections between neurons. This idea forms the basis of learning-related synaptic plasticity—the idea that memories are stored as changes in the number and strength of synapses between neurons—a framework that has endured as a model for understanding the biology of memory.

Studies of memory in rodents and goldfish performed in the 1960s and 1970s demonstrated that long-term memory required protein synthesis whereas short-term memory did not [35]. Subsequent studies of learning-related plasticity in organisms ranging from *Aplysia* sensory-motor synapses to rodent hippocampal synapses similarly demonstrated that long-lasting forms of plasticity can be differentiated from short-term plasticity by their dependence on RNA and protein synthesis [36]. Molecular biological approaches led to the identification of many genes that contribute to long-term plasticity and memory and to the elucidation of specific patterns of neuronal activity and specific signaling pathways that trigger changes in RNA and protein synthesis within neurons. These findings gave rise to the idea that activity-dependent changes in the neuronal transcriptome and proteome mediate and/or maintain the changes in neuronal structure and physiology that result in persistent changes in synaptic strength.

Initially, studies of gene expression underlying memory focused on activity-dependent changes in transcription in the nucleus. The discovery of immediate early genes, such as c-fos, arc, and zif268, that were induced during memory formation implied that the neuron was the unit of long-term plasticity and memory [37]. Immediate early genes are now widely used to map neurons involved in memory formation, in line with the idea that memories are encoded within networks of discrete neurons in the brain.

In contrast to this neuron-centric view of memory formation, studies of learning-related plasticity revealed that plasticity was synapse-specific, that is, it could occur at some but not all synapses made by an individual neuron [38]. This finding raised questions about how the products of gene expression, synthesized in the nucleus, could be targeted to alter structure and function at subsets of synapses within a single, highly polarized and compartmentalized neuron. One solution to this question came from findings that mRNAs localized to distal dendrites and synapses [39], where their translation could be regulated by activity. These findings included detection of polyribosomes at the base of synapses as well as the identification of a subset of mRNAs that localized to distal dendrites. Studies from several labs have identified hundreds to thousands of dendritically localized mRNAs [40–42] translation of which could alter the structure and function of synapses, thereby making the synapse (or neighborhood of synapses), rather than the entire neuron, the unit of plasticity.

**Fig. 2:**
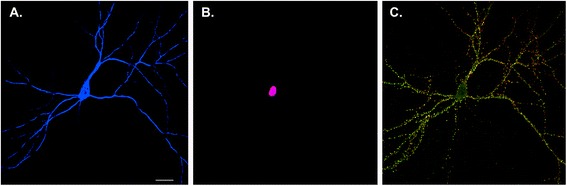
Neuron versus synaptic maps of memory. The Golgi method used by Ramon y Cajal at the turn of the 19th century revealed that the brain consisted of individual nerve cells, leading to the formulation of the neuron doctrine. In modern neurobiology, expression of soluble fluorescent proteins allows visualization of individual neurons in the brain or, as shown here, in a hippocampal pyramidal neuron in dissociated culture (*blue*) (**a**). Identification of immediate early genes, such as cFos, provides a means of labeling the nuclei of individual neurons that are activated to undergo transcription following neuronal activity (*violet*) (**b**). This provides a neuron-level map of activity. However, each neuron forms thousands of synapses, shown in **c** by labeling a single neuron with presynaptic (*green*) and postsynaptic (*red*) markers (*yellow* where the pre- and post-synaptic elements are adjacent to one another). Note that this neuron is connected to other neurons in the culture that are not shown. Developing a synaptic rather than a neuron-wide map of memory requires obtaining activity-dependent markers that label the activated synapses rather than nuclei. Understanding synaptic maps will not only require understanding the identity of individual neurons but also the details of their cell-to-cell interactions

Local translation at synapses has been shown to regulate translation in a synapse-specific manner [43, 44] and inhibiting local translation at synapses in a variety of in vitro preparations has been shown to block long-term learning-related plasticity [45, 46]. In one of these preparations, the *Aplysia* sensory-motor culture system, we directly tested the relationship between transcriptional regulation in the nucleus and translational regulation at the synapse during synaptic plasticity and found that stimulus-induced newly transcribed localized mRNAs were delivered throughout the neuron but were only translated at locally stimulated synapses [47]. The implication of this finding is that activity-dependent transcription sets the entire neuronal arbor in a state of readiness to respond to local cues via regulated translation of localized mRNAs. From the perspective of activity-dependent gene regulation, this idea shifts the focus from the neuron as the unit of plasticity to the synapse (or neighborhoods of synapses). In so doing, it underscores the need to refine the current neuron-based maps of memories by developing synaptic maps of memory in the brain (Fig. 2).

The focus on the synapse as the unit of plasticity calls into question aspects of the neuron doctrine. Thus, while the neuron doctrine assumes that neurons are separate, autonomous units, cell biological studies of synapses indicate that all components of the synapse are intimately interconnected, including not only the pre- and post-synaptic compartments but also glial processes. For example, in one study of local translation at *Aplysia* sensory-motor synapses, we found stimulus-induced local translation of a reporter depended on trans-synaptic signals from the post-synaptic motor neuron compartment to the presynaptic sensory neuron compartment [44]. Taking the idea of non-cell autonomy to an extreme, recent studies have reported that neurons and/or glia can transfer mRNAs and microRNAs to neighboring neurons and/or glia [48]. This finding raises the provocative idea that gene expression can be regulated by transfer of genetic information from cell to cell. As such, the idea of “local” translation serving to alter the structure and function of synapses encoding a memory suggests that “synaptic maps” of memory are not encoded by discrete synaptic compartments of individual cells but rather by complex fluid synaptic neighborhoods. These neighborhoods include not only the products of gene expression that are made in the nucleus of the neuron the synapse belongs to but also signals (including RNAs) from neighboring neurons and glia.

To add an additional layer of complexity onto this view of memory, single cell RNA sequencing experiments have uncovered remarkable complexity in the nerve cells and glia within the brain [49]. These findings indicate that synaptic neighborhoods are not just comprised of common modules of pre-synaptic, post-synaptic, and glial components but that they also exist in a multitude of distinct flavors or “ethnicities” as neighborhoods. A recent electron microscopic reconstruction of a small volume of mouse neocortex revealed that the formation of synapses between neurons is more dependent on cell identity than on proximity [50]. The diversity of cell identities, in combination with the idea that cell identity drives synapse formation, indicates that generating a synaptic map of memory will require understanding the distinct identities of the participating cells as well as the details of their cell-to-cell interactions.

As a cell biologist interested in understanding memory, the challenges moving forward include identifying conserved local processes that persistently alter synaptic function and developing methods to manipulate these processes in order to test their function in the formation and storage of memory. Possibilities include local mechanisms of translational regulation at synapses, trans-synaptic signaling pathways (including the transfer of RNAs via extracellular vesicles), interactions between synaptic cell-adhesion molecules, and even extracellular matrix dynamics. As an example of the latter, Roger Tsien has proposed that memories may be stored in the pattern of holes formed within the perineuronal net, a specialized extracellular matrix structure that is formed at the end of critical periods in the brain [51]. Taken together, recent lessons learned suggest that elucidation of these local processes requires consideration of the synaptic compartment as a local environment rather than as a collection of separate and autonomous membrane bound compartments. While morphology, from Golgi stains to electron microscopy, emphasizes the boundaries between cells in the brain, molecular cell biological studies uncover a fundamental role for local cell–cell interactions and interconnections in the encoding of memories.

## Memory as a functional consequence of epigenetic priming in engram neurons

### Andrii Rudenko and Li-Huei Tsai

Memory formation, storage, and recall constitute the essence of human nature. The search for the mechanisms underlying learning and memory has revealed the importance of a number of molecular and cellular processes, such as activity-dependent gene expression, intracellular signaling cascades, and synaptic plasticity [52, 53]. The long-lasting attempts to characterize memory localization recently resulted in identification of specific neuronal populations—so-called engrams—that provide a physical location for the storage and retrieval of memory traces [14, 15, 54, 55]. Despite discovery of the engram cells, molecular mechanisms of memory storage remain unclear. We propose that epigenetic alterations taking place in these cells may represent a critical process involved in the long-term retention of memory traces.

One well-studied example of such alterations is histone acetylation, a covalent mark of active chromatin. Mutations in CBP, a gene product necessary for the acetylation of multiple memory genes, result in severe intellectual disability in humans as well as in mice [56–58]. Conversely, histone deacetylase inhibitors (HDACi) were shown to restore histone acetylation and ameliorate cognitive deficits in a CBP-deficient mouse model [56, 57]. Moreover, HDACi have been found to ameliorate memory deficits in mouse models of Alzheimer’s disease [59, 60]. In addition to histone acetylation, several other epigenetic mechanisms, including DNA methylation and hydroximethylation, have also been demonstrated to regulate memory function [61, 62].

While inhibiting HDACs was found to be effective in enhancing synaptic plasticity and memory, such inhibition per se, in the absence of neural stimulation, produced very limited results [63, 64]. Thus, HDAC inhibition appears to convey its effects on learning and memory via facilitating gene expression elicited by neural activity, a phenomenon known as epigenetic priming [65, 66].

As discussed earlier in this Forum by Pignatelli, Ryan, and Tonegawa, a recent study by Ryan et al. [16] has elegantly demonstrated that inhibiting protein synthesis during the memory consolidation window does not disrupt memory retrieval by means of engram activation. This exciting observation suggests that while augmented synaptic strength may be critical for memory encoding, some other mechanisms, potentially involving epigenomic modifications in engram neurons, appear to be necessary for memory trace storage. The recent discovery that long-term memory can be re-instated following erasure of its synaptic expression strongly supports this idea [67]. We propose that, mechanistically, the engram cells are marked, or tagged, not only synaptically [68] but also at the epigenetic level, be it histone acetylation, methylation, DNA methylation, or Topoisomerase IIβ-dependent topological changes of the DNA/chromatin, as recently suggested by our work [62, 69]. Specifically, the initial phase of memory formation would cause changes in the epigenetic state of the engram cells through a priming event which may be protein synthesis-independent (for example, epigenetic modifications to make specific genomic regions poised for efficient transcriptional activation). Such changes may also lead to long-lasting alterations in chromatin structure and function underlying the memory consolidation process. Finally, memory retrieval would signal the engram cells potentiating initial epigenetic priming, including molecular events such as generation of DNA breaks within the promoter areas of early response genes such as *c-Fos*, *Npas4*, *Nr4a1*, and *Egr1* [69], triggering expression of the primed genes leading to protein synthesis and increases in the number and strength of the synapses. Such a chain of events may explain why, even after considerable neurodegeneration, HDAC inhibition coupled with behavioral training is capable of reinstating learning and retrieval of long-term, and even remote, memory [59, 70]. This scenario may be possible if engram cells, epigenetically primed by the initial learning experience and capable of re-engaging in the chain of molecular events leading to memory retrieval, still remain in the brain after neurodegeneration.

We should note that in these early days of functional memory engram investigation, we still do not have satisfactory answers to many important questions. For example, the exact molecular and structural features of engram-containing networks, or potential mechanisms that might allow participation of a specific neuron in different engram ensembles, currently remain unknown. Moreover, although there is accumulating evidence of the epigenetic marks/tags that would prime engram cells for an efficient transcriptional response, the exact nature of those marks remains unclear. Deciphering such priming signatures will help us immensely in understanding the mechanistic basis of memory processing.

## Memory mechanisms: LTP and LTD in partnership, not merely in opposition

### Richard W. Tsien, Gord Fishell, and Caitlin Mullins

How memory is stored in the brain can be effectively queried by examining how this process is affected in neuropsychiatric conditions. To this end, many aspects of the emerging pathophysiology of autism spectrum disorders (ASD) may provide insight into the tuning of synaptic strength in memory [71]. In ASD and related intellectual disability (ID), gene discovery points to dysregulation of interrelated neuronal functions, including control of nuclear gene expression, local protein synthesis in dendrites, and excitation:inhibition (E:I) coordination. These functions are linked together in feedback loops involving electrical or chemical sensors, termed “homeostats”. The feedback loop may malfunction as a result of disease-causing mutations in any of the components. Given the effects of these disorders on cognition, it is likely no accident that the same set of functions loom large in current thinking about memory. Indeed, consideration of ASD and other neuropsychiatric diseases provides fresh perspective on the basic underpinnings of memory. From this viewpoint, we offer some thoughts about the relationship between LTP and LTD and the way that information in the brain may be stored and retrieved.

**Fig. 3:**
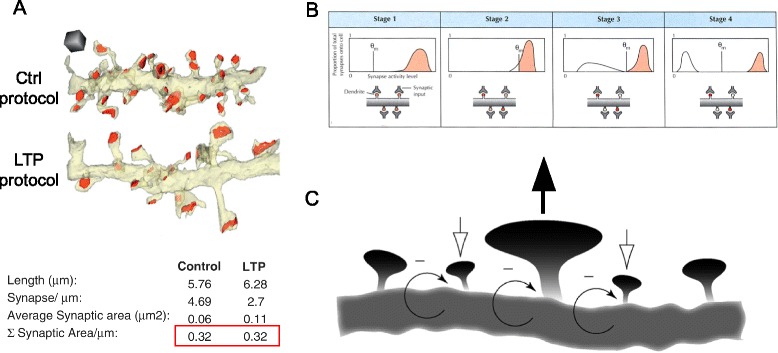
Possible mechanisms of synaptic modification in memory storage. **a** Structural synaptic scaling is analyzed by serial section electron microscopy in hippocampal tissue under control and LTP (induced via theta burst stimulation) conditions. Control and LTP dendrites have equal postsynaptic areas (*red outline*) despite differences in synaptic density and size. Adapted from [72], with permission. **b** Representation of metaplasticity occurring, shifting the plasticity threshold (*θ*_*m*_). The graphs on the *top row* depict the distribution of synaptic responses, *red shading* indicating LTP and *white shading* LTD. The four synapses illustrated (*bottom row*) reflect how the shifting of θ_m_ would impact the strength of individual synapses (intensity of *red shading* correlating to strength). Stage 1 depicts a saturating event, where all of the synaptic strengths have been excited to levels above θ_m_. Stage 2 shows a metaplastic response to the activity levels reached in stage 1. In stage 2, θ_m_ has shifted such that some synapses are below the new threshold and weaken in stage 3. This changed θ_m_ leads to a new stable state, illustrated in stage 4. Reproduced from [75], with permission. **c** Synaptic potentiation at one spine (center, *upward-pointing black arrow* added for emphasis) is predicted to temporarily increase activity at adjacent spines. In response to the increased activity, homeostatic plasticity weakens the entire area (*negative feedback arrows*), with closely neighboring spines being disproportionately weakened (*downward-pointing white arrows*). The weakened spines allow the overall dendritic length to maintain a constant level of activity with the central spine still maintaining a level of potentiation. Adapted from [76], with permission

In an intriguing study published in 2012, Bourne and Harris used serial section electron microscopy to compare dendrites receiving either theta burst LTP or control stimulation [72]. As expected for LTP [34, 73], they found single spines with increased postsynaptic area by 2 h. Surprisingly, however, this occurred concomitant with a remarkable reduction in the number of small spines, leaving the total postsynaptic area per unit dendritic length unchanged compared with control (Fig. 3a). The constancy of total postsynaptic area per unit dendritic length fits well with prevailing concepts about homeostasis and normalization, but it also raises provocative questions about the neurobiological mechanism and organization of memory.

Two previously proposed and rather different hypotheses considered here might explain the underlying basis of Bourne and Harris’ observations. In reviewing evidence for a “sliding threshold” concept [74], synaptic plasticity with flexible rules (metaplasticity), Deisseroth et al. [75] discussed a scheme that could fit with Bourne and Harris’ results (Fig. 3b). If a stretch of dendrite receiving strong inputs undergoes LTP but then autoregulates the threshold dividing LTD and LTP, it would attain a new equilibrium that fractionates the synapses into stronger and weaker subpopulations (Fig. 3b). Also pertinent to Bourne and Harris’ results, Rabinowitch and Segev [76] focused on lateral coordination of synaptic strength, invoking unknown mechanisms of local regulation on dendritic branches. In their hypothesis, LTP at spines receiving strong synaptic input is yoked together by a compensatory mechanism that weakens, or even eliminates, nearby neighbors (Fig. 3c).

Although these papers make no reference to each other, they converge on a shared view of synaptic plasticity, jointly supported by biological evidence and theoretical rationale: a synapse may undergo Hebbian strengthening or weakening as an individual entity but, over time, it can also behave as a connected entity, operating in coordination with other synapses in the same neuron or dendritic branch. Further, LTP and LTD can cooperate to redistribute synaptic weight. This notion differs from the traditional analogy between synapses and digital information storage devices, in which bits are stored and retrieved independently. On the other hand, coordination amongst multiple synapses, made by different inputs, provides benefits with regard to issues of normalization and signal-to-noise.

What can be said about the molecular mechanism(s) that might allow or even drive the coexistence of LTP and synaptic weakening in close proximity along a stretch of dendrite? This question has received much less attention than strengthening and weakening of the same synapses (see, for example, an elegant demonstration of LTP and LTD in direct opposition [22]). In the spirit of this Forum, we list here multiple possibilities for such lateral interaction:

1. Concentration of the excitatory neurotransmitter glutamate ([Glu]) must fall off with increasing distance from a strong input and could play some role in lateral interactions. Whereas NMDA receptors are essential for most forms of postsynaptically expressed LTP and are driven by high [Glu], lower levels of [Glu] would be sensed by the more sensitive metabotropic glutamate receptors (mGluR) at nearby synapses and could foster LTD mechanisms.
2. Different programs of local protein synthesis may be triggered by NMDARs and mGluRs and support LTP (e.g., increased synthesis of AMPA receptors) or LTD (e.g., upregulation of Arc). As we propose elsewhere [71], mutual inhibition between such translational programs could help enforce a sharp threshold dividing LTP and LTD, whereby local signals mediate LTP proximally and LTD at a distance. Interestingly, defects in either program can give rise to ASD.
3. βCaMKII (beta calcium/calmodulin-dependent protein kinase II) is well-suited to serve as a local sensor of activity-dependent rises in Ca^2+^ because of its high Ca^2+^/CaM sensitivity. Hence, βCaMKII can also serve as an arbiter, dictating the decision between exo- and endocytosis of AMPARs to promote LTP/LTD. High [Ca^2+^] activates βCaMKII, increasing AMPAR exocytosis [77]. In contrast, low [Ca^2+^] leads to CaM-free, kinase-deactivated βCaMKII interacting with Arc, facilitating AMPAR endocytosis [78].
4. AMPA receptors can also be redistributed along the dendritic length by coordination of exocytosis of AMPARs and LTP at one site with endocytosis of AMPARs and LTD in flanking regions. A frank lateral transfer of AMPARs [79] could support a coupling of LTP/LTD at nearby dendritic spines.

Many studies on the cell biology of the dendrite take on different meaning if considered in this light. Also, an organizational principle of “robbing Peter to pay Paul” might engender marked strengthening of some synapses (rich getting richer) at the expense of others (poor getting poorer), thus contributing to empirical observations of a highly skewed distribution of synaptic weights [80–82]. It remains to be seen, however, whether the observations of Bourne and Harris are relevant to behavioral memory in vivo and whether the tradeoff of synaptic strength is predominantly a local, dendritic branch-based phenomenon as suggested by Rabinowitch and Segev [76] or also strongly dependent on regulation at the neuron-wide level [75, 83]. We have spoken little about the temporal dimension but the static snapshots in Fig. 3 are clearly shorthand for complex dendritic dynamics. Whatever the molecular/subcellular mechanism and dynamics, tradeoffs in synaptic strength will have a strong influence on the way that memory traces are stored and retrieved. As the debate continues about memory engrams and persistent changes at the level of synapses, synaptic neighborhoods, ensembles of cells, and even whole circuits, we would do well to consider redistribution in net synaptic strength as an underlying mechanism, rather than merely net increases or decreases. Perhaps we should modify silicon-based notions of memory units with independent read-write capabilities and embrace assemblies—not just “cell assemblies” [1, 17] but also “synaptic assemblies”—in their full spatiotemporal glory.

## A role for adult-born neurons in pattern separation

### J. Tiago Gonçalves, Matthew Shtrahman, Stephen T. Johnston, and Fred H. Gage

Memory involves the complex interplay between forming representations of novel objects or events and developing generalizations of similar experiences. Distinct instances of similar events must be discriminated—for example, being able to find your car at work despite parking in a different spot every day—but, at the same time, experiencing just a fragment of a familiar experience, such as a particular smell, can trigger specific memories from your childhood. The interplay between forming distinct memories and generalizing events is conceptualized to involve two separate processes: pattern separation and pattern completion.

The dentate gyrus (DG) is the information gateway of the hippocampal formation and as such plays a crucial role in hippocampal function, including the formation of episodic memories. Additionally, the DG is one of only two regions within the mammalian brain that generates new neurons throughout the life span of an individual in both rodents and humans [84–86]. There is increasing evidence that adult-born dentate granule cells (DGCs) are important for fine pattern separation of similar but distinct events [87, 88].

#### The DG as a pattern separator

The elegantly delineated anatomy of the hippocampus has long served as a substrate for theories about memory formation. It is grossly delineated into a series of interconnected loops within four major subregions. Specifically, the DG of the hippocampus receives excitatory input from the entorhinal cortex (EC) via the perforant path [89]. The DG then projects through the mossy fiber tract to form powerful synaptic connections with CA3 neurons, which in turn project to CA1, forming the classic trisynaptic pathway. In addition, the DG projects to and receives input from local inhibitory interneurons, most notably from the hilus.

In rodents, the DG contains approximately four- to fivefold more neurons than up- or downstream EC and CA3, respectively. Thus, input from relatively few cells is processed by a much larger neural network within the DG before generating a condensed output. However, only a small percentage of DGCs is activated in response to a given event [90, 91]. Based on these characteristics, modeling studies postulate that the DG is a competitive network that can function as a pattern separator by partially de-correlating inputs [3, 92]. One prediction from this theory is that the DG is critical for forming memories of events that are similar but not identical to each other. This prediction is supported by accumulating evidence from both high-resolution functional MRI studies in humans and studies of “behavioral pattern separation” in rodents [93, 94], where subjects discriminate between similar environments or sensory stimuli presented at different times.

#### What mechanistic role do adult-born DGCs play in behavioral pattern separation?

Although the gross anatomical connectivity of the DG suggests a role in pattern separation, the DG’s ability to perform this function appears to be further enhanced via its ability to incorporate new neurons. Adult-born DGCs undergo a lengthy process of morphological and physiological maturation before they fully integrate into the local hippocampal network [95]. Each immature DGC enters a critical period of greater plasticity approximately 4 to 6 weeks after it is born. These immature DGCs exhibit greater excitability [96], receive less inhibition from local interneurons [97], are more broadly tuned to input stimuli [98], and exhibit greater synaptic plasticity [99] than mature cells. Therefore, immature adult-born DGCs may perform unique computational tasks critical for hippocampal function, in particular behavioral pattern separation [87, 100].

**Fig. 4:**
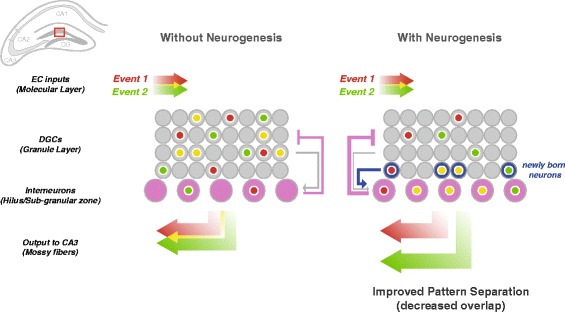
Immature adult-born neurons improve pattern-separation in the DG by enhancing feedback inhibition. Two events are encoded in separate but partially overlapping populations of activated DGCs in the DG (*red* and *green*, with overlap in *yellow*). DGCs receive strong inhibitory inputs from interneurons (*purple*) in the hilus and sub-granular zone. It is hypothesized that hyperactive immature adult-born DGCs (*blue*) drive these interneurons, enhancing feedback inhibition from the hilus, which results in decreased overlap of activated DGCs and output to CA3, thereby improving pattern separation

The physiological properties of immature neurons at the single-cell level, however, might yield counterintuitive predictions at the circuit level. While hyperexcitability and broad tuning of immature neurons may allow the hippocampus to encode novel stimuli, their broad tuning would also seem to suggest that a hippocampal circuit rich in immature neurons would fire more frequently and indiscriminately to various environmental inputs. Yet, this explanation appears to conflict with the role of the DG in pattern separation. Therefore, it has recently been proposed that, though hyperexcitable themselves, these immature neurons’ key contributions could be to suppress overall DG activity, maintain network sparseness, and thereby decrease interference between memory representations [101–103] as illustrated in Fig. 4. Individual immature neurons may be more excitable and broadly tuned but it is hypothesized that these cells in turn activate inhibitory interneurons in the DG and hilus, resulting in greater inhibition of mature DGCs. Developing a unified picture of how single-cell physiological features combine to determine circuit-level responses and behavior presents a great challenge for future work.

#### Future steps for understanding the role of adult-born neurons in the DG

Previous efforts to link DG function to cellular and molecular mechanisms have been hindered by technical limitations. First, although extracellular recording of DGCs has been used to monitor the dynamics of neuronal firing patterns, sparse activity within the DG makes it difficult to locate neurons, identify neuronal subtypes, and monitor the activity of a large population of DGCs [104]. Second, immunohistochemical analysis of immediate early gene expression, such as c-fos, can assess activity across a large population but only at limited time points, providing a limited snapshot of DG activity. Nevertheless, advances in in vivo recording techniques promise to eventually enable the monitoring of DG cells during behaviorally relevant tasks. In vivo imaging methods [105] in particular would be perfectly suited for recording DG activity—hundreds of cells can be monitored simultaneously and cellular subtypes identified through the use of genetic markers. Combined with modern techniques to manipulate activity in adult-born neurons, imaging large populations of neurons in vivo would permit testing the hypothesis that immature and mature DGCs work cooperatively to maintain the normal sparse network activity of the DG. These methods also have the potential to provide further insights into the mechanisms behind memory formation and recall, revolutionizing our understanding of the features encoded by both mature and immature DGCs and of how responsiveness to those features changes with learning.

## Mechanisms for memorizing temporal sequence and interval

### Mu-ming Poo and Yang Dan

The temporal sequence and interval of events are essential elements of episodic and procedural memories. Where and how sequence and interval information is stored in the brain remains a mystery. If modifications of synaptic connectivity are the cellular substrates for memory storage, then the challenge is to understand how distributed synaptic changes within neural circuits represent the sequence and interval of previously experienced sensory or motor events.

In his physiological postulate for perceptual memory, Hebb [1] proposed that reverberating activity generated by perceptual experience along distinct neuronal pathways could provide temporary storage of the experience. Repeated firing of a specific assembly of neurons in a particular temporal sequence could then serve to imprint the memory by modifying synaptic connections among the cells and re-activation of the assembly in this sequence represents recall of the memory.

The popular version of Hebb’s postulate refers to synaptic modifications based on correlated pre- and postsynaptic firing (“cells that fire together wire together”), and it received experimental validation with the discovery of long-term potentiation (LTP) [5] and long-term depression (LTD) [106] induced by high- and low-frequency presynaptic stimulation, respectively—high-frequency stimulation results in postsynaptic spiking and thus LTP due to correlated activity, whereas low-frequency stimulation fails to do so, leading to LTD. The discovery of spike timing-dependent LTP and LTD [107, 108] led to further revision of Hebb’s learning rule—the sequence of pre- and postsynaptic spiking, rather than simple coincidence of activity, is critical for determining whether the synapse is strengthened or weakened. The defined time windows for spike timing-dependent plasticity (STDP) [8, 109, 110] and its presence at many excitatory synapses [111] suggest that temporal information may be stored via STDP, and Hebb’s assembly could be established by sequence-dependent synaptic strengthening or weakening. In addition to the well-studied sequence replay in the hippocampus (see contribution by Long and Buzsáki in this Forum), this hypothesis was supported by a finding that repetitive visual stimulation with a unidirectional moving spot that evokes sequential spiking of the cortical neurons enhances their sequential firing in response to a flashed stimulus, in a manner that depends on the speed of the conditioning stimulus and activation of NMDA receptors in the visual cortex [112].

The time window for STDP, normally quite narrow (~20 ms) for LTP and wider and more variable (~20–100 ms) for LTD, also imposes a limit on the time intervals between pre- and postsynaptic spiking that can leave a long-term imprint at the synapse. Could the interval be extended via polysynaptic excitation within a network? A simple test of this idea was performed in a random synaptic network formed by dissociated hippocampal neurons in culture. Repetitive pair-pulse stimulation at a fixed interval was applied to a single input neuron and changes in the efficacy of polysynaptic connections within the network were measured. The result showed that intervals up to a few hundred milliseconds could lead to distributed long-term modifications (both LTP and LTD) of polysynaptic pathways within the network [113]. While this study provides proof of principle for encoding long intervals through polysynaptic delays, does the natural neuronal network in the brain contain serially connected groups of synchronously firing neurons (synfire chain) [114] to encode long sequences and temporal intervals? The finding of sequential firing of neurons in the premotor area HVC of songbirds during each song syllable (which lasts >100 ms [115]) indicates the existence of synfire chains in the brain, the formation of which underlies song learning [116]. Spike sequences lasting for several seconds have also been observed in the mammalian neocortex [117].

In addition to STDP in polysynaptic networks, memories of time intervals on the order of seconds are also likely to involve other mechanisms. In a study in zebrafish larvae, Sumbre et al. [118] applied a unidirectional moving visual stimulus repeatedly at a fixed temporal interval (several seconds) and recorded the population activity of tectal neurons with Ca^2+^ imaging. They found that following cessation of the visual stimulation, sequential firing of the tectal neurons resembling that evoked by the moving stimulus reappeared at the same time interval as the conditioning stimuli, and this rhythmic reappearance lasted for up to 20 s. This post-conditioning spontaneous rhythmic firing of tectal neurons reflects short-term memory of a specific rhythm with time intervals of seconds. Interestingly, since the tectal activity did not persist continuously through the several seconds of interval, it is unlikely mediated by reverberation of activity in the polysynaptic network. The underlying circuit mechanism and the location of memory storage in this case remain unknown.

Remembering the association between events separated by intervals ranging from seconds to tens of seconds has been referred to as temporal associative learning, and has been found in recent studies to depend critically on entorhinal cortex (EC)–hippocampal circuits [119–121]. Pathway-selective inhibition and optogenetic activation of the EC–hippocampal circuit showed that layer II “island cells” and layer III neurons of the EC control temporal associative learning [119, 120]; in particular, persistent activity of medial EC layer III neurons may play a key role [121]. Recent studies have also shown the existence of hippocampal “time cells” that fire at particular moments in a temporally structured experience [122–124], suggesting a function that parallels that of place cells in spatial memory. The sequential activation of such time cells in the hippocampus may reflect temporally structured inputs from the cortex and other brain regions or alternatively firing chain generated within local hippocampal circuits by repetitive sequential activity-induced strengthening of synaptic connections.

Theories and models of sequence learning and interval timing have been proposed [125–127] but very few studies have directly addressed the circuit and synaptic mechanisms [128, 129]. Since the cellular and synaptic building blocks of memory are increasingly well characterized and the technology for manipulating specific cells is becoming available (see other contributions to this Forum), the storage mechanisms for sequence and interval information are now amenable to fruitful exploration. Understanding these mechanisms is a pre-requisite for further studies of circuit mechanisms underlying higher cognitive functions involving complex temporal information processing such as human language, the ultimate challenge to neuroscience.

## Hippocampal sharp wave-ripple: a repetitive mechanism to support single trial learning

### John Long and György Buzsáki

Memories are not “imprinted” immediately but evolve over time. Memory has many forms and supportive brain mechanisms. While learning complex skills and habits, such as walking elegantly in high-heel shoes or riding a unicycle, may require tens to thousands of repetitions, consciously remembering episodic information, such as recalling one’s first date, often requires only a single trial. The brain can achieve such a feat by deploying a mechanism that repeats segments of the original episode subconsciously hundreds to thousands of times after the experience. Such repetitions occur during non-attentive, off-line states of brain operation in the form of hippocampal sharp wave-ripples (SPW-Rs). SPW-Rs operate as a time-compressing mechanism, which can transfer information from the hippocampus to numerous regions of the neocortex. SPW-Rs also serve as a pre-conscious “mixer” of existing knowledge and recently acquired information. Thus, acquiring episodic memory is a two-stage process, initiated by a rapid encoding mechanism during attentive waking and followed by a protracted consolidation process during “off-line” states of the brain [130].

#### SPW-Rs are self-generated hippocampal patterns

The SPW-R complex is the most synchronous and phylogenetically preserved pattern in the mammalian brain, associated with enhanced transient excitability in the hippocampus and its partner structures. These super-synchronous bursts arise when the release of subcortical neuromodulators within the hippocampus is decreased—for example, during consummatory behaviors and slow wave sleep. A SPW-R is a superposition of two events, the sharp wave-related population burst (SPW), which emerges in the strongly recurrent system of the CA2/3 regions, and the fast ripple oscillation, which is dominant in the CA1 output circuit of the hippocampus. The synchronous discharge of CA3 pyramidal cells excites primarily the mid-apical dendrites of the CA1 region and the inward currents brought about by this transient depolarization process (40–150 ms) manifest extracellularly as the local field potential SPW. The response of target circuits to the strong SPW-related depolarization is a fast oscillatory balancing act between principal cells and perisomatic inhibitory interneurons, resulting in the LFP ripple and the phase-locked discharge of sequentially active neurons [130]. It is this waking history-dependent, sequential firing of a large fraction of hippocampal cells, and consequent recruitment of neocortical neurons, that makes SPW-Rs a candidate biomarker for memory consolidation.

#### Compressed replay of experience during SPW-Rs serves memory

**Fig. 5:**
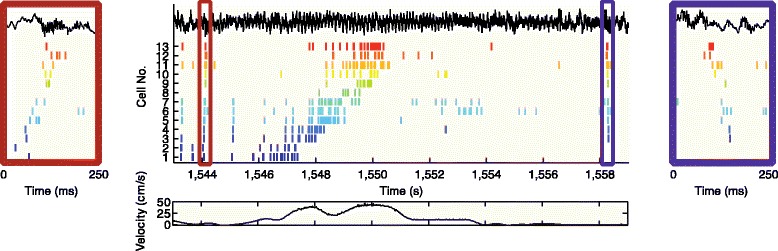
Place cell sequences experienced during behavior (*middle panel*) are replayed in both forward (*left panel*) and reverse (*right panel*) direction during awake SPW-R. The rat is moving from left to right on a familiar track. Spike trains for place fields of 13 CA3 pyramidal cells (*color ticks*, spikes of individual neurons) on the track are shown before (forward replay; *left red box*), during (*middle*), and after (reverse replay; *right blue box*) a single traversal. The CA1 local field potential is shown on top (*black traces*) and the animal’s velocity is shown below. Reproduced from [137]

The restructuring of hippocampal–cortical networks through synaptic plasticity is necessary for the formation of new episodic memories. Neurons participating in SPW-R events are organized to fire sequentially and the orderly structure of these events reflects a temporally compressed version of the sequential neuronal firing patterns observed in the waking animal [131, 132] (Fig. 5; “replay”). For example, the sequences of “place cells” [133] in a novel environment are formed from a combination of relatively fast-firing and slow-firing groups of pyramidal neurons. Conspicuously, the former neurons exhibit relatively unchanging temporal dynamics while the latter are highly plastic. The greater plasticity of slow-firing pyramidal neurons is evidenced by their greater gain in place specificity during maze exploration and their increased SPW-R-related recruitment during sleep following waking experience [134]. These sequences are not confined to the hippocampus–entorhinal system; the SPW-R output also brings about sequential activations in neocortical circuits, such as the prefrontal cortex [135], leading to systems-level consolidation of the memory trace. Even more direct evidence for the role of SPW-R sequences in memory consolidation is provided by close-loop truncation of SPW-Rs. Selective elimination of the hundreds of SPW-R-assisted replays of recently learned sequences during post-learning sleep prevented rats from becoming proficient at a spatial-reference memory task [136]. This supports the hypothesis that single-trial learning is made possible by repeated off-line SPW-R replays of the wake-experienced episode.

#### Memory and planning: retrospective and prospective roles for SPW- R

A particularly striking feature of SPW-Rs is that the neuronal sequences contained in them can propagate both forward and backward (Fig. 5) relative to waking experience. Before the beginning of a journey upon a familiar maze, the upcoming sequences are preplayed during SPW-Rs in a forward manner; that is, prior to a run, the CA1 pyramidal cells fire in a sequence consistent with the upcoming trajectory of the animal in the maze. At the end of the journey, again, the same neurons are reactivated during SPW-Rs but now in a reversed direction as the hippocampus recapitulates the landmarks passed by the animal, though in a time-compressed manner. These findings support the hypothesis that forward replay events play a role in “planning” upcoming trajectories [137]. This hypothesis is further supported by several recent experiments that demonstrate that the routes chosen by the animal in two-dimensional environments or between maze corridors can be predicted by the spike sequence content of SPW-Rs [138]. The subconscious route-priming role of SPW-Rs is also supported by other experiments, which show that truncating SPW-Rs prior to a choice results in a spatial working memory deficit [139]. Because the neurons participating in SPW-Rs sequences are drawn from a diverse pool of log-scale firing rate distributed neurons, with varying coding, biophysical, circuit, and plasticity properties, these events can transit a vast array of preexisting and new information to downstream cortical partners [134].

To date, memory mechanisms in experimental animals are typically studied in the framework of spatial navigation [133]. However, it is important to emphasize that mechanisms of memory and planning have evolved from mechanisms of navigation in the physical world. Therefore, neuronal algorithms underlying navigation in real and mental space, as well as place memory and episodic memory, are fundamentally the same [140].

## Where is the study of Hebbian memory mechanisms going?

### Charles F. Stevens

*BMC Biology* brought together some of the leaders in the learning and memory field to identify, from eight separate perspectives, some of the currently most important questions and approaches for understanding memory formation, consolidation, and retrieval. My job is to relate the current state of the field to how, in my opinion, it might evolve.

With rare, and highly valued, exceptions, a field develops organically from where it started and from the questions that grew out of each advance along the way. Although several of the contributions to this Forum relate key ideas from earlier eras (Cajal, Golgi, and Semon) to modern views, my starting point for the questions that drive learning and memory research will be Hebb’s 1949 “firing - > wiring” proposal [1] for learning (cited in the introduction to this Forum and in four of the contributions).

Although Hebb’s book was always well known to psychologists, it had essentially no impact on neurophysiology (as neuroscience was known in those days) for about a quarter of a century because the longest lasting form of synaptic plasticity known before 1973 (the date of Bliss and Lomo’s LTP paper [5]) was post-tetanic potentiation (PTP) and this form of plasticity lasted, at most, only a few minutes. The only suggested brain mechanism for memory storage had been “reverberating circuits” and Von Neumann gave a compelling argument against the plausibility of this idea in his 1957 lectures at Yale on “The computer and the brain” [141]. Neurophysiologists were all very interested in the cellular basis of memory—everyone agreed it was a central problem—but no one had any idea how to study it.

As with any novel finding at odds with current knowledge and concepts, Bliss and Lomo’s LTP paper [5] did not make an immediate splash. Indeed, during the next decade only a hand-full of researchers worked on LTP. But over the succeeding decades the study of LTP grew explosively to become a large and important sub-field of neuroscience, some would even say a preoccupation. As this Forum shows, we have learned an enormous amount about synaptic plasticity in the two-thirds of a century since Hebb’s *The Organization of Behavior*. But where will we go now?

First, I need to confess that my record for predicting the future is abysmal: I have failed close to 100 % of the time. This time, however, I am trying a new strategy to see if I can move up from abysmal to, say, just terrible. This new approach is to see where the questions that drive our research have come from and then ask what we might have missed in asking these questions. Because of space constraints, I will focus on a single question: what might Hebbian learning be missing?

Although Behaviorism is pretty much dead [142], there is absolutely no question that reinforcement plays a key role in what behaviors an animal selects. And, as anyone who has experienced a traumatic event can tell you, the circumstances associated with the event are committed to an enduring memory (what were you doing when you heard about the 9/11/2001 terrorist attacks in the United States?). Of course, everyone is aware of this, yet the focus on Hebb’s rule tends to neglect these other types of learning. Major exceptions to this are the climbing fibers in the cerebellum and the mossy fibers in the CA3 region of the hippocampus, where a single impulse or brief burst will fire the target neuron and is believed to potentiate the other synapses that are active at the same time. For both of these cases, Hebb’s rule is sufficient to determine what needs to be learned.

So my specific prediction is that future scientists working on LTP/LTD (or STDP) will also include reward (positive or negative) related mechanisms in their investigations of synaptic plasticity at the molecular, cellular, circuit, and behavioral levels.

Where can we look to get guidance on wedding Hebb’s rule to reinforcement learning? One of the simplest and best studied examples of alternative learning rules is the fruit fly olfactory system, where flies can learn to approach or avoid any odor if that odor is paired with reward (sugar water) or punishment (electric shock). A good example from Glenn Turner’s laboratory [143] is an electrophysiological study showing how a specific dopamine neuron can decrease the synaptic strength of inputs into a specific mushroom body output neuron (MBON). The inputs into the MBON are synapses from 2000 mushroom body Kenyon cells and each odor activates a specific subpopulation of the Kenyon cells, about 100/2000. These 100 cells constitute a *tag* for that odor [144], a unique subpopulation of Kenyon cells whose firing stands in for the odor’s combinatorial code generated by odorant receptor neurons in the fly’s antennae. When the odor is paired with a shock, the specific dopamine neuron whose synapses overlap with the MBON’s dendritic arbor and with the Kenyon cell’s axonal arbor is activated. Firing of this dopamine neuron is what causes the Kenyon cell synapses to weaken and this change in synaptic strength is responsible for the avoidance of the odor associated with punishment.

Most scientists who study LTP/LTD/STDP use mammals as their experimental animals. It seems, however, that the dopamine system is, to a large degree, evolutionarily conserved even though the fly circuits are generally less complex than the corresponding ones in vertebrates. I am not aware of a review article that documents the insect/vertebrate parallels in the dopamine systems but the literature on the amygdala, a vertebrate center for valence learning, is vast [145] and can be compared to the large fly literature devoted to mushroom body learning and memory [146].

We can see over the next few years if my success for predicting future trends in science has improved.
